# Alarming prevalence of *Candida auris* among critically ill patients in intensive care units in Dhaka City, Bangladesh

**DOI:** 10.1017/ash.2023.222

**Published:** 2023-09-29

**Authors:** Fahmida Chowdhury, Kamal Hussain, Sanzida Khan Khan, Dilruba Ahmed, Debashis Sen, Zakiul Hassan, Mahmudur Rahman, Sajeda Prema, Alex Jordan, Shawn Lockhart, Meghan Lyman, Syeda Mah-E-Muneer

## Abstract

**Background:** *Candida auris* is a multidrug-resistant yeast capable of invasive infection with high mortality and healthcare-associated outbreaks globally. Due to limited labratory capacity, the burden of *C. auris* is unknown in Bangladesh. We estimated the extent of *C. auris* colonization and infection among patients in Dhaka city intensive care units. **Methods:** During August 2021–September 2022 at adult intensive care units (ICUs) and neonatal intensive care units (NICUs) of 1 government and 1 private tertiary-care hospital, we collected skin swabs from all patients and blood samples from sepsis patients on admission, mid-way through, and at the end of ICU or NICU stays. Skin swab and blood with growth in blood-culture bottle were inoculated in CHROMagar, and identification of isolates was confirmed by VITEK-2. Patient characteristics and healthcare history were collected. We performed descriptive analyses, stratifying by specimen and ICU type. **Results:** Of 740 patients enrolled, 59 (8%) were colonized with *C. auris*, of whom 2 (0.3%) later developed a bloodstream infection (BSI). Among patients colonized with *C. auris*, 27 (46%) were identified in the ICU and 32 (54%) were identified from the NICU. The median age was 55 years for *C. auris*–positive ICU patients and 4 days for those in the NICU. Also, 60% of all *C. auris* patients were male. Among 366 ICU patients, 15 (4%) were positive on admission and 12 (3%) became colonized during their ICU stay. Among 374 NICU patients, 19 (5%) were colonized on admission and 13 (4%) became colonized during their NICU stay. All units identified *C. auris* patients on admission and those who acquired it during their ICU or NICU stay, but some differences were observed among hospitals and ICUs (Figure). Among patients colonized on admission to the ICU, 11 (73%) were admitted from another ward, 3 (20%) were admitted from another hospital, and 1 (7%) were admitted from home. Of patients colonized on admission to the NICU, 4 (21%) were admitted from the obstetric ward, 9 (47%) were admitted from another hospital, and 6 (32%) were admitted from home. In addition, 18 patients with *C. auris* died (12 in the ICU and 6 in the NICU); both patients with *C. auris* BSIs died. **Conclusions:** In these Bangladesh hospitals, 8% of ICU or NICU patients were positive for *C. auris*, including on admission and acquired during their ICU or NICU stay. This high *C. auris* prevalence emphasizes the need to enhance case detection and strengthen infection prevention and control. Factors contributing to *C. auris* colonization should be investigated to inform and strengthen prevention and control strategies.

**Figure:**
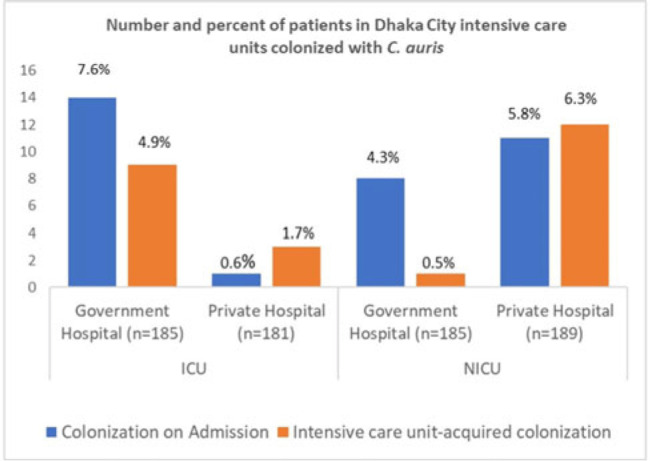

**Disclosure:** None

